# Benefits, barriers and opinions on multidisciplinary team meetings: a survey in Swedish cancer care

**DOI:** 10.1186/s12913-018-2990-4

**Published:** 2018-04-05

**Authors:** Linn Rosell, Nathalie Alexandersson, Oskar Hagberg, Mef Nilbert

**Affiliations:** 1 Institute of Clinical Sciences, Division of Oncology and Pathology, Lund University, Scheelev. 2, 223 63 Lund, Sweden; 2 Regional Cancer Centre South, Region Skåne, Sweden; 3 Clinical Research Centre, Copenhagen University Hospital, Hvidovre, Denmark; 4 Danish Cancer Society Research Center, Copenhagen, Denmark

**Keywords:** Tumor board, Cross-sectional study, Health care survey, Multidisciplinary team conference, Patient preferences

## Abstract

**Background:**

Case review and discussion at multidisciplinary team meetings (MDTMs) have evolved into standard practice in cancer care with the aim to provide evidence-based treatment recommendations. As a basis for work to optimize the MDTMs, we investigated participants’ views on the meeting function, including perceived benefits and barriers.

**Methods:**

In a cross-sectional study design, 244 health professionals from south Sweden rated MDTM meeting structure and function, benefits from these meetings and barriers to reach a treatment recommendation.

**Results:**

The top-ranked advantages from MDTMs were support for patient management and competence development. Low ratings applied to monitoring patients for clinical trial inclusion and structured work to improve the MDTM. Nurses and cancer care coordinators did less often than physicians report involvement in the case discussions. Major benefits from MDTM were reported to be more accurate treatment recommendations, multidisciplinary evaluation and adherence to clinical guidelines. Major barriers to a joint treatment recommendation were reported to be need for supplementary investigations and insufficient pathology reports.

**Conclusions:**

Health professionals’ report multiple benefits from MDTMs, but also define areas for improvement, e.g. access to complete information and clarified roles for the different health professions. The emerging picture suggests that structures for regular MDTM evaluations and increased focus on patient-related perspectives should be developed and implemented.

**Electronic supplementary material:**

The online version of this article (10.1186/s12913-018-2990-4) contains supplementary material, which is available to authorized users.

## Background

Multidisciplinary team meetings (MDTMs) have widely been implemented in cancer care based on the principle that interdisciplinary case discussions lead to improved treatment recommendations based on updated and evidence-based knowledge or expert opinion. The MDTM structure is broadly considered to improve communication, coordination and decision making [1]. The MDTM is part of the weekly clinical duties for most physicians, nurses and coordinators in cancer care, links clinical information from various sources and represents a pivotal point of the patient care pathway. Benefits from MDTMs relate to improved care processes, adherence to clinical and up-to-date treatment recommendations, which have been documented in several cancer types [2–8]. Other potential benefits include shorter lead times, increased attention to patient-related perspectives, competence development, training opportunities for younger colleagues and the possibility to identify patients eligible for clinical trials [8]. Studies on the relation between MDTM, quality of care and survival have reached different conclusions, potentially explained by differences in study design, MDTM format, case selection and different diagnoses studied [2, 4, 6, 7, 9]. Core MDTM expertise varies between diagnoses, but typically includes surgeons, medical oncologists, radiation therapists, radiologists and pathologists. More recently, experts in nuclear medicine and molecular pathology, contact nurses, research nurses and cancer care coordinators have been added to the multidisciplinary team. Greater multidisciplinarity is, however, not necessarily associated with more effective decision-making and treatment implementation [9].

A number of issues will influence the benefit from a MDTM, e.g. participation from qualified and effective experts, case selection, access to relevant information, discussion format and structure, leadership, health professionals’ interactions, technical equipment and administrative processes [9, 10]. Most MDTMs are held on a weekly basis, though a recent focus on shorter lead times and efficient diagnostic processes in Swedish cancer care has led to biweekly meetings in select diagnoses and hospitals. A growing number of MDTMs are video-based with regional or national participation. Though there is general agreement of the value of MDTM, the structure is also questioned since it is resource-demanding due to  an increasing cancer incidence, participation from a growing number of experts, increased meeting frequency to grant timely treatment, evaluation based on refined diagnostic methods and more complex treatment algorithms. Information on the structure and function of individual MDTMs is scarce and a wide variety of meeting standards has been documented [4, 9, 11]. To provide a basis for structured and targeted improvements in cancer care, we investigated health professionals’ views on MDTMs, including perceived benefits from MDTM-based recommendations and barriers to reach joint recommendations, with correlation to discipline, profession, hospital type and diagnostic area.

## Methods

We performed a cross-sectional study on health professionals’ views of MDTMs. Data was based on an electronic survey that was distributed to all identified participants in the 50 MDTMs in the south Sweden health care region. This region has a population of 1.8 million and provides specialized cancer care services provided by one University hospital and six county hospitals. These 50 MDTMs were initially identified in a study on the determinants of MDTM costs, which has recently been presented and documents a mean MDTM duration of 0.88 h, mean 12.6 cases discussed and a mean cost per case discussion of 212 (range 91–595) EUR [12]. The MDTM meetings were held on a weekly basis and included 19 meetings at local hospitals and 31 meetings at the University hospital. Of the 50 MDTMs, 19 were video-based regional and two were video-based national MDTMs with participation from health professionals from other hospitals. A list of MDTM participants was provided by the cancer care coordinators at each hospital. All participants in these 50 MDTMs were eligible for the study. A small number (< 10) individuals participated in more than one weekly MDTM and were assigned to the predominant diagnosis and meeting base on impact, i.e. from leading the meeting or from the number of case discussions. In total, 362 participants were identified to whom study invitations accompanied by a link to the electronic survey were distributed by e-mail.

We constructed an electronic survey (Surveymonkey.com) with three parts; a first part with five demographic questions and information on weekly MDTM participation times, a second part where the informants were asked to rate 20 statements on MDTM structure and function and a third part where the informants were asked to prioritize up to three possible benefits from MDTM and up to three potential barriers for shared MDTM recommendations (Additional file 1) . The questionnaire was constructed by the research group, was in Swedish and the contents related to benefits, barriers and choice of statements were largely collected from previous publications in the field. Data on validity and reliability are not available, but prior to data collection the questionnaire underwent pilot testing in five MDTM participants from various disciplines and professions. The demographic questions included data on age, sex, profession (physician vs nurse/coordinator), hospital type (county vs university hospital) and discipline (surgery, medicine, radiology, pathology). Surgery included general surgery, urology, thoracic surgery, neurosurgery, vascular surgery, orthopedic surgery and gynecology. Medicine included medical oncology and radiation oncology, pediatric oncology, hematology, pulmonology, endocrinology and neurology. Radiology included radiology, nuclear medicine and clinical physiology. Cancer care coordinators represent a new role in Swedish health care with responsibilities for booking and coordinating diagnostic and therapeutic procedures. Since the number of coordinators was low, this group was analyzed together with the nurses.

Health professionals’ views on MDTMs were evaluated based on 20 statements that the respondents were asked to rate on a seven-point Likert scale from 1 (strongly disagree) to 7 (strongly agree) with a possibility to answer “do not know/not applicable”. The statements referred to the participants’ individual competence and their roles at the MDTM (*n* = 3), functional aspects of the conference, e.g. guidelines for referral and documentation, technology, availability of relevant information (*n* = 11) and overall impact from MDTM recommendations e.g. perceived benefits for patient management, education and training, clinical study inclusion and use of resources (*n* = 6). To collect information on perceived benefits and barriers, the respondents were asked to select the three out of 13 most important benefits of MDTMs and the three out of 15 most important barriers to reaching a joint treatment recommendation. Respondents who provided one to three responses were considered in the further analyses. The alternatives defined in the survey were selected from previous publications [9–11, 13–15]. All data are available from the corresponding author upon request.

The response profiles were analyzed in relation to professions, disciplines, hospital types and cancer-specific MDTMs. All statistical analyses were performed in R, version 3.2.2 [16]. Benefits of MDTMs and barriers to reaching a joint recommendation were analyzed using chi squared tests with significance set at *p* = 0.05. Data on opinions of MDTMs based on Likert scale data are presented in a diverging stacked bar chart and were analyzed using chi squared tests. Bonferroni correction was applied to correct for multiple testing.

## Results

Complete responses that allowed for further analyses were obtained from 244 of 362 (67%) MDTM participants. Further analysis of non-responders was not possible due to lack of data on this subset. Of the respondents, 56% were women. The age distribution was 2% in the age group 20–29 years, 13% 30–39 years, 33% 40–49 years, 33% 50–59 years and 19% of the respondents were ≥ 60 years of age. Of the respondents, 70% were physicians and 28% were nurses and coordinators. Discipline was surgery in 47%, medicine in 29%, radiology in 14% and pathology in 7%. Hospital type was 52% university hospital and 48% county hospitals. The respondents represented teams from various cancer diagnoses: 27% gastrointestinal and hepatobiliary cancer, 21% breast cancer and malignant melanoma, 19% urological and gynecological cancer, 19% lung cancer and 12% other tumors, i.e. head and neck tumors, CNS tumors, sarcomas and endocrine tumors.

**Fig. 1:**
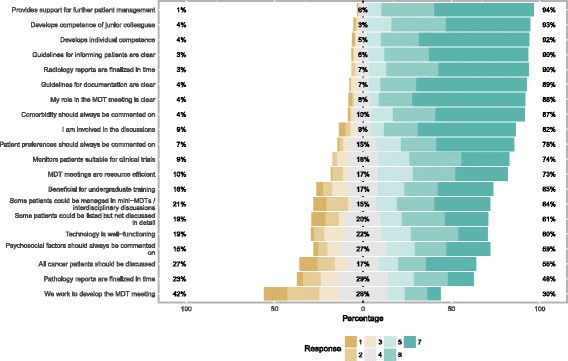
Respondents’ views on MDTMs. Diverging stacked bar chart demonstrating the frequency of different levels of agreement on 20 statements. Scores from 1 (strongly disagree) to 7 (strongly agree)

The respondents’ views on MDTMs are summarized in Fig. 1. Overall, affirmative scores were given to the majority of the statements. Agreement (scores 5–7) was particularly strong for *provides support for further patient management* (94%), *develops competence of junior colleagues* (93%) and *develops individual competence* (92%). The two issues that received the lowest fraction of affirmative responses were *pathology reports are finalized in time* (48%) and *we (*i.e. *the MDT) work to develop the MDTM* (30%). The responses were consistent without major differences in relation to profession, discipline, hospital type or cancer-specific MDT. No significant differences applied for scores 1–3, whereas minor differences were identified for the affirmatory responses. Nurses and coordinators did more often than physicians agree to *MDTM being resource efficient* (88% vs 69%, *p* = 0.008) and *all cancer patients should be discussed* at MDTMs (74% vs 49%, *p* = 0.0015), but did less often report *being involved in the discussions* (57% vs 90%, *p* = 0.0005). The views also differed between cancer-specific MDTMs related to whether *all cancer patients should be discussed* in MDTMs, which was supported by a majority of members in teams working with breast cancer, GI cancer and other tumors (53–78%), but to a lesser extent in teams working with lung cancer and urological-gynecological cancer (31–38%) (*p* = 0.0005).

**Fig. 2:**
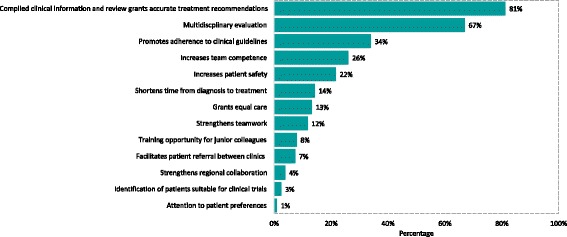
Benefits of MDTMs. Respondents were asked to choose the reasons they considered most important, maximally three. Percentages refer to the total number of respondents (*n* = 203)

Analyses on the most important benefits of MDTM were based on answers submitted by 203 respondents. The two predominant benefits were *compiled clinical information and review results in more accurate treatment recommendations* (81%) and *multidisciplinary evaluation* (67%), followed by *promotes adherence to clinical guidelines* (34%), *increases team competence* (26%) and *increases patient safety* (22%) (Fig. 2). The two reasons that were the least selected were *attention to patient preferences* (1%) and *identification of patients suitable for clinical trials* (3%). Perceived benefits of MDTM differed between various health care profession, discipline and hospital type, but was not influenced by the cancer field served (Table 1). Nurses and coordinators more often than physicians (28% vs 9%, *p* < 0.001, significant after Bonferroni correction) considered *shortened time from diagnosis to treatment* as a major benefit of MDTM. Pathologists did more often than physicians of other disciplines refer to *strengthens teamwork* (43% vs 7–11%, *p* = 0.005). Health professionals working in university hospitals did more often than those employed at county hospitals report *increases team competence* as a major benefit of MDTM (34% vs 19%, *p* = 0.015), whereas professionals in county hospitals more often selected *multidisciplinary evaluation* (75% vs 59%, *p* = 0.026).

**Table 1:** Reported benefits of MDT meetings in relation to health profession, hospital type, discipline and cancer type being discussed

	Frequency of all responses	Nurses/ coordinators	Physicians	*P*-value	University hospital	County hospital	*P*-value	Pathology	Radiology	Medicine	Surgery	*P*-value	Breast cancer	Lung cancer	GI cancer	Uro/Gyn cancer	Other tumors	*P*-value
Compiled clinical information and review grants accurate treatment recommendations	81%	75%	83%	0.221	78%	84%	0.381	100%	86%	78%	81%	0.278	76%	77%	88%	74%	96%	0.120
Multidisciplinary evaluation	67%	70%	66%	0.732	59%	75%	**0.026**	57%	68%	69%	67%	0.880	63%	63%	71%	64%	74%	0.830
Promotes adherence to clinical guidelines	34%	30%	35%	0.512	36%	32%	0.550	29%	46%	29%	33%	0.449	35%	29%	36%	31%	39%	0.897
Increases team competence	26%	26%	26%	1.000	34%	19%	**0.015**	14%	21%	27%	27%	0.702	22%	37%	21%	33%	22%	0.327
Increases patient safety	22%	19%	23%	0.699	24%	20%	0.628	29%	25%	13%	25%	0.274	24%	26%	19%	21%	17%	0.924
Strengthens teamwork	12%	11%	12%	1.000	13%	10%	0.535	43%	7%	9%	11%	**0.005**	13%	11%	14%	13%	4%	0.833
Training opportunity for junior colleagues	8%	2%	10%	0.073	5%	10%	0.194	0%	0%	15%	8%	0.075	4%	11%	7%	10%	9%	0.781
Shortens time from diagnosis to treatment	14%	28%	9%	< 0.001*	18%	11%	0.240	14%	21%	9%	14%	0.504	13%	14%	14%	18%	13%	0.973
Grants equal care	13%	17%	12%	0.474	13%	13%	1.000	7%	7%	18%	14%	0.494	17%	20%	10%	13%	4%	0.401
Facilitates patient referral between clinics	7%	11%	6%	0.212	4%	10%	0.096	0%	4%	11%	8%	0.442	15%	3%	5%	8%	0%	0.095
Strengthens regional collaboration	4%	0%	5%	0.116	3%	5%	0.719	0%	0%	9%	3%	0.100	2%	6%	2%	3%	13%	0.138
Identification of patients suitable for clinical trials	3%	6%	1%	0.121	0%	5%	0.050	7%	0%	2%	3%	0.559	2%	0%	3%	5%	0%	0.649
Attention to patient preferences	1%	4%	0%	0.056	1%	1%	1.000	0%	0%	0%	2%	0.727	0%	0%	0%	3%	4%	0.217

*P*-values < 0.05 in bold writing

^*^P-values that remain significant after applying Bonferroni correction (*p* < 0.004)

**Fig. 3:**
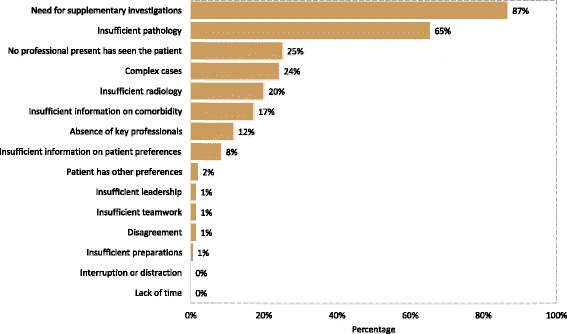
Barriers to joint recommendations in MDTMs. Respondents were asked to choose the barriers they considered most common, maximally three. Percentages refer to the total number of respondents (*n* = 216)

Analyses of the most common barriers to reaching a joint recommendation were based on answers submitted by 216 respondents. The predominant barriers were *need for supplementary investigations* (87%) and *insufficient pathology* (65%), followed by *no professional present has seen the patient* (25%), *complex cases* (24%) and *insufficient radiology* (20%) (Fig. 3). *Patient preferences, insufficient leadership, insufficient teamwork, disagreement, insufficient preparations,* *interruption or distraction  *and *lack of time* were rare causes, reported by 0–2% of the respondents. Reported barriers differed between professions, hospital types, disciplines and cancer-specific MDTMs (Table 2). Physicians did  more often than nurses and coordinators (29% vs 13%, *p* = 0.024) refer to *no professional present has seen the patient*. *Complex cases* were reported by 37% of physicians in medicine compared to 29% of pathologists, 20% of surgeons and 14% of radiologists (*p* = 0.049). *Complex cases* were also more often referred to by professionals in the university hospital than in county hospitals (33% vs 17%, *p* = 0.005). Health care  personnel at the university hospital did more often than personnel in county hospitals refer to *absence of key professionals* (17% vs 7%, p = 0.04). In contrast, health professionals in county hospitals more frequently chose *insufficient pathology* (73% vs 56%, *p* = 0.015) and *no professional present has seen the patient* (31% vs 18%, *p* = 0.024). Minor differences were observed between the cancer-specific MDTMs related to *no professional present has seen the patient*, which was rarely identified in the breast cancer teams (*p* = 0.002, significant after Bonferroni correction), and *disagreement* on the recommendations, which was more commonly reported from members in urological and gynecological MDTM teams (*p* = 0.015) (Table 2).

**Table 2:** Reported barriers to joint recommendations from MDT meetings in relation to health profession, discipline and hospital type

	Frequency of all responses	Nurses/ coordinators	Physicians	*P*-value	University hospital	County hospital	*P*-value	Pathology	Radiology	Medicine	Surgery	*P*-value	Breast cancer	Lung cancer	GI cancer	Uro/ Gyn cancer	Other tumors	*P*-value
Need for supplementary investigations	87%	94%	84%	0.069	87%	86%	0.841	93%	86%	88%	85%	0.812	85%	87%	90%	84%	85%	0.921
Insufficient pathology	65%	72%	63%	0.247	56%	73%	**0.015**	36%	62%	72%	66%	0.083	79%	72%	56%	58%	62%	0.102
No professional present has seen the patient	25%	13%	29%	**0.024**	18%	31%	**0.024**	14%	31%	25%	25%	0.699	6%	23%	29%	42%	27%	0.002*
Complex cases	24%	22%	25%	0.854	33%	17%	0.005*	29%	14%	37%	20%	**0.049**	23%	28%	19%	23%	35%	0.585
Insufficient radiology	20%	20%	20%	1	16%	23%	0.172	21%	21%	22%	18%	0.95	17%	10%	19%	30%	19%	0.232
Insufficient information on comorbidity	17%	11%	19%	0.21	15%	19%	0.485	14%	10%	13%	22%	0.354	19%	26%	20%	9%	8%	0.193
Absence of key professionals	12%	17%	10%	0.218	17%	7%	**0.040**	14%	17%	5%	14%	0.256	15%	10%	12%	9%	12%	0.94
Patient has other preferences	2%	4%	1%	0.576	4%	0%	0.055	0%	0%	2%	3%	0.864	2%	3%	0%	5%	0%	0.486
Insufficient information on patient preferences	8%	6%	9%	0.585	9%	8%	0.835	14%	10%	5%	9%	0.627	4%	8%	8%	12%	12%	0.742
Disagreement	1%	2%	1%	1	1%	2%	1	0%	0%	2%	2%	1	0%	0%	0%	7%	0%	**0.015**
Insufficient teamwork	1%	2%	1%	1	2%	1%	0.616	7%	0%	0%	2%	0.209	2%	0%	2%	2%	0%	1
Insufficient leadership	1%	2%	1%	1	1%	2%	1	0%	0%	3%	1%	0.576	0%	3%	0%	2%	4%	0.568
Insufficient preparations	1%	2%	0%	0.253	1%	0%	0.478	0%	0%	0%	1%	1	0%	0%	2%	0%	0%	1
Interruption or distraction	0.%	0%	0%		0%	0%		0%	0%	0%	0%		0%	0%	0%	0%	0%	
Lack of time	0.%	0%	0%		0%	0%		0%	0%	0%	0%		0%	0%	0%	0%	0%	

*P*-values < 0.05 in bold writing

^*^P-values significant after applying Bonferroni correction (*P* < 0.003)

## Discussion

Health professionals who participate in cancer-related MDTMs report an overall positive attitude, but also identify key issues for improvement, which fits with reports from other health care systems [2, 4]. MDTMs are typically chaired by physicians and more recent inclusion of nurses and coordinators in the meetings has been reported to improve team performance [17]. We identified differences between physicians and nurses/coordinators related to the estimated impact from MDTM on time to treatment, resource-efficiency and involvement in the case discussions. Nurses and coordinators did more often (28% vs 14%) refer to MDTMs contributing to shorter time to treatment, which may reflect that nurses and coordinators who participate in MDTM may immediately plan and book further procedures and treatments. Whereas the views on development of individual competence did not differ between physicians and nurses/coordinators, the latter group reported being less involved in the case discussions. An observing rather than an interacting role of nurses in MDT meetings has been reported also by other investigators with reports that the medical perspectives dominate over care perspectives during MDTMs [18, 19]. An important aspect of improvement of MDTMs relate to an appropriate skill mix of a multidisciplinary team and development and implementation of MDTM structures and procedures [20]. These observations suggest that the roles of nurses and coordinators should be highlighted to improve MDTM function. Responsibilities that could be targeted to nurses include consideration of comorbidity, psychosocial aspects, rehabilitation and supportive care needs, patient preferences and relevant clinical trials [19]. Cancer care coordinators could take responsibility for all relevant documentation being available prior to the case discussions [18, 20].

Though many MDTM groups struggle with how to best include patient-related perspective in the decision process, a limited focus on these aspects have been documented in several studies [21]. Restivo et al. found that psychological, socio-demographic and relational aspects were discussed in 30% of the cases and patient’ preferences were discussed in 10% of the cases at MDTMs in French health care [22]. Divergent treatment priorities between physicians and patients have been demonstrated in multiple studies and cancer types. If the MDTM aims to contribute to individualized treatment decisions and implementation of the MDTM’s recommendations, patient values and preferences need to be considered. Our data demonstrate that in Swedish health care 78% of health professionals agree that patient preferences should be commented on during the MDTMs, but only 1% of the respondents identify patient perspectives as a major benefit from MDTMs (Figs. 1 and 2). The need to consider comorbidities was supported by 87% of the respondent and 17% considered comorbidity to be a major barrier for a joint MDTM recommendation (Figs. 1 and 3). Leadership and interactions between the MDTM participants are central in this process. MDTM leaders often express a clear view on the optimal treatment recommendation. Team members may counteract this by providing additional patient-related information, which may influence the further discussion, though perhaps  based on fragmented and selected information [22]. Additionally, when the information is conveyed to the patient, it needs to be balanced, which requires that controversies and differences in opinion have been clarified. Current observations suggest that though the premises of multidisciplinary care involve addressing patients’ needs, routines for how this should be granted at the MDTM need to be developed and will likely require substantial revision of the current meeting structure [9, 23–26].

The MDTM may be a suitable and relevant time point to consider patients for inclusion into clinical trials. In our data, 74% of the respondents supported that the MDTM could be used for this purpose, but only 3% identified this as a key benefit of MDTMs. Training for multidisciplinary teams in communication around clinical studies has been implemented and evaluated in the UK with promising results related to ease of communication and understanding of the impact for trial inclusion [27].

The two most important benefits from MDTMs were reported to be  treatment recommendations based on compiled clinical information and multidisciplinary evaluation, followed by adherence to guidelines, increased team competence and patient safety (Fig. 2). Reference to increased competence and strengthened team work fits well with data from an international survey that report that seeking advice on treatment recommendation and participation in multidisciplinary discussion were the main reasons for MDT attendance [28]. The MDTM may also improve communication, positively influence the work environment and is an important part of continuous medical education [28]. In our study, MDTMs were considered more valuable for training of younger colleagues/residents (93%) than for education of undergraduate students (65%) (Fig. 1). Health professionals at the university hospital did more often (34% vs 19%) than their colleagues in local hospitals refer to the MDTM contributing to an improved team competence. Pathologists did significantly more often (43% vs 7–11%) than other disciplines refer to teamwork as an important benefit of the MDTM, which most likely reflects differences in working cultures between pathologists, who independently diagnose cases, and other disciplines, where teamwork is part of the everyday clinical work.

Failure to reach a joint recommendation has been reported to occur in 6–52% of case discussions during MDTMs [16, 28]. Considering the increasing demand for meeting time, efforts to reduce this figure are important. The main barriers to reach a joint recommendation identified in our study were need for supplementary investigations and insufficient pathology, followed by no professional present who had seen the patient and complex cases (Fig. 3). Absence of key professionals was more frequently (17% vs 7%) reported from the university hospitals than the county hospitals, which may reflect a vulnerable access to highly specialized competences. The participants rated compiled clinical information as one of the most important benefits from MDTM, but at the same time identified insufficient clinical information as a main barrier for a joint recommendation, which is supported also by observations from other health care systems [16, 29]. Though poor leadership, insufficient teamwork, disagreement and time pressure were by the respondents identified as less important, other studies have documented that factors such as poor leadership, insufficient teamwork, disagreement and time pressure as barriers for efficient MDTM recommendations [17, 21]. MDTM case reviews have been shown to change the initial treatment plan in up to a third of the cases, with the highest likelihood in complex cases [22, 28, 30, 31]. In our study, complex cases were more often (33% vs 17%) identified as barriers for recommendations by MDTM members at the university hospital compared to county hospitals. This difference likely reflect case selection and underscores the need for highly specialized competences for high-quality case evaluations and the need for the MDTM team to define core competences and support these members in improvement initiatives related to efficient decision-making.

Guidelines for which patients should be discussed at MDTMs should regularly be reviewed since the benefit of multidisciplinary evaluation and the need for core expertise likely differs between cancer types, tumor subsets, disease stages and patient subgroups. Of the respondents, 61–64% were positive to targeted approaches, e.g. listing of standard cases without detailed discussion or mini-MDTMs with selected disciplines present. Alternative case discussion formats were in our study supported by teams in lung cancer and urological and gynecological cancer and support for prioritization has in previous studies been gained from e.g. urological and colorectal cancer [13, 30]. Though data on the use of mini-MDTM are scarce, this principle has been suggested to be time and resource saving compared to full MDTMs [28, 31].

Only 30% of the respondents reported work to develop the MDTMs, though use of e.g. independent observers or evaluation instruments have been shown to change case management and improve MDTM quality. Several instruments have been developed and have performed favorably related to validity and interrater reliability [11, 32–34]. Work to optimize MDTM recommendations need to consider the MDTM function as well as the implementation rate of the recommendations made with careful consideration of shortcomings and differences in views between the participants [9].

Strengths of the study include a population-based approach with participation from all MDTMs in our health care region, a 67% response rate and a large sample size, which allows for subgroup-specific analyses in relation to professions, disciplines, hospital type and cancer-specific MDTs. Weaknesses relate to our development of a questionnaire the results of which cannot readily be compared to other studies. The perceived benefits and barriers to MDTMs were largely restricted to issues previously identified in scientific studies. Use of select statements and predefined benefits and barriers risks overseeing less common perspectives, although the informants could provide free text comments. Furthermore, since standardized MDTM improvement programs have not been implemented in Sweden, the input from health professionals could not be studied in relation to whether the MDTM in question was well-functioning or not.

## Conclusions

Health professionals in Swedish cancer care are overall positive to MDTMs, but also identify several shortcomings. Nurses and coordinators report being less active in the case discussions. MDTMs are rarely used to screen patients for inclusion into clinical trials. The focus on patients-related perspectives and preferences is weak. Only one-third report structured work to evaluate and improve the MDTM function. Considering the increasing needs for MDTM and the considerable resources invested, these observations call for implementation of regular MDT evaluations and further research on how to best improve MDTM efficacy.

### Practical implementation

Health professionals report strong benefits from MDTM related to support for further patient management and professional competence development and identify issues for improvement that include finalized pathology reports prior to the meeting and implementation of structured work to improve MDTM function. Nurses and cancer care coordinators did more often than physicians perceive that the meetings were resource efficient, but did less often than physicians report being involved in the case discussions. Predominant MDTM benefits were compiled clinical information and review, multidisciplinary evaluation and adherence to clinical guidelines. Major barriers to reach a joint treatment recommendation were the need for supplementary investigations and insufficient pathology. These issues would be valuable to consider in future MDTM improvement programs.

## Additional file

Additional file 1: Questionnaire. Electronic survey distributed by e-mail to MDTM participants (*n* = 362). (PDF 315 kb)

## Abbreviations

MDTM: Multidisciplinary team meeting
