# Supplementary material for: Benefits, barriers and opinions on multidisciplinary team meetings: a survey in Swedish cancer care
Source: BMC Health Serv Res. 2018 Apr 5;18:249. doi: 10.1186/s12913-018-2990-4 (PMC5887214; doi:10.1186/s12913-018-2990-4)
Supplement: Supplementary file 1 — Questionnaire. Electronic survey distributed by e-mail to MDTM participants (n = 362). (PDF 315 kb) [file 12913_2018_2990_MOESM1_ESM.pdf]

Purpose

During 2016 Regional Cancer Centre South hade an assignment to work for equal access to multidisciplinary team meetings (MDTM). We have identified you as a member of a MDT through mapping the MDTM in the south health care region.

The purpose is to increase the knowledge, investigate MDT member's experiences and collect data on resources spent. We kindly ask you to fill in this questionnaire which takes 5-10 minutes. Received answers will be anonymously handled and all participants will get the opportunity to take part of the final rapport.

Please accept our thanks in advance!
***Mef Nilbert*, Head of Operations, Regional Cancer Centre South**
***Linn Rosell*, Health Care Development Officer, Regional Cancer Centre South**

Questions about yourself and the time spent on MDTM

1. Age;

2. Gender;

- ○ Male
- ○ Female
- ○ Wish not to define

3. Profession;

- ○ Resident physician
- ○ Specialist physician
- ○ Nurse
- ○ Secretary/coordinator
- ○ None of the above, please define (e.g. assistant physician, social worker, physiotherapist, occupational therapist);

4. If you work as a nurse, do you have any of the assignment listed below?

- ○ Contact nurse
- ○ Research nurse
- ○ None of the above, please define;

5. Specialisation;

- [ ] Surgery (including urology, orthopaedics, thoracic surgery, gynaecology)
- [ ] Medicine (including respiratory medicine, haematology, oncology, neurology)
- [ ] Radiology (including nuclear medicine, clinical physiology)
- [ ] Pathology/cytology
- [ ] None of the above, please define;

6. Country council where I work;

- [ ] Region Skåne
- [ ] Region Kronoberg
- [ ] Region Halland
- [ ] Blekinge county council
- [ ] None of the above, please define;

7. How many MDTMs do you participate weekly?

- [ ] 1
- [ ] 2
- [ ] ≥ 3
- [ ] Don´t know

8. What type of MDTM do you participate in? (More than one alternative can be marked)

- [ ] Local
- [ ] Regional
- [ ] National
- [ ] None of the above, please define;

9. How much time do you spend on MDTM per week regarding; (Specify in half and full hours)

Preparation? ____

Participation? ____

Post-MDTM work? ____

10. Do you find the time allocated to MDTM to be sufficient?

- ☐ Yes
- ☐ No
- ☐ Don´t know
- ☐ If no, please motivate;

____

Questions regarding aim and implementation of MDTM

11. Which reasons for MDTM do you consider most important? (Choose maximum **3** alternatives)

- ☐ Complied clinical information and review grants accurate treatment recommendations
- ☐ Facilitates patient referral between clinics
- ☐ Multidisciplinary evaluation
- ☐ Grants equal care
- ☐ Shortens time from diagnosis to treatment
- ☐ Promotes adherence to clinical guidelines
- ☐ Attention to patient preferences
- ☐ Increases team competence
- ☐ Strengthens teamwork
- ☐ Identification of patients suitable for clinical trials
- ☐ Training opportunity for junior colleagues
- ☐ Increases patient safety
- ☐ Strengthens regional collaboration
- ☐ Are there other benefits of MDTM you find of value? Please define;

12. My role at the MDTM; (The answers are ranked from 1-7, where **1**=strongly disagree and **7**=strongly agree)

	1 (=strongly disagree)	2	3	4 (=partly agree)	5	6	7 (=strongly agree)	Don't know/not relevant
My role in the MDTM is clear	○	○	○	○	○	○	○	○
I am involved in the discussions	○	○	○	○	○	○	○	○
Develops individual competence	○	○	○	○	○	○	○	○

Questions regarding MDTM structure and guidelines

13. My opinion on benefit, structure and guidelines related to MDTM; (The answers are ranked from 1-7, where **1**=strongly disagree and **7**=strongly agree)

	1 (=strongly disagree)	2	3	4 (=partly agree)	5	6	7 (=strongly agree)	Don't know/not relevant
Guidelines for documentation are clear	○	○	○	○	○	○	○	○
Guidelines for informing patients are clear	○	○	○	○	○	○	○	○
We work to develop the MDTM	○	○	○	○	○	○	○	○
Technology is well-functioning	○	○	○	○	○	○	○	○
Pathology reports are finalized in time	○	○	○	○	○	○	○	○
Radiology reports are finalized in time	○	○	○	○	○	○	○	○
Provides support for further patient management	○	○	○	○	○	○	○	○
MDTM are resource efficient	○	○	○	○	○	○	○	○
Beneficial for undergraduate training	○	○	○	○	○	○	○	○
Develops competence of junior colleagues	○	○	○	○	○	○	○	○
Monitors patients suitable for clinical trials	○	○	○	○	○	○	○	○

14. My opinion regarding patients being discussed at MDTM; (The answers are ranked from 1-7, where **1**=strongly disagree and **7**=strongly agree)

	1 (=strongly disagree)	2	3	4 (=partly agree)	5	6	7 (=strongly agree)	Don't know/not relevant
Comorbidity should always be commented on	◯	◯	◯	◯	◯	◯	◯	◯
Psychosocial factors should always be commented on	◯	◯	◯	◯	◯	◯	◯	◯
Patient preferences should always be commented on	◯	◯	◯	◯	◯	◯	◯	◯
All cancer patients should be discussed	◯	◯	◯	◯	◯	◯	◯	◯
Some patients could be listed but not discussed in detail	◯	◯	◯	◯	◯	◯	◯	◯
Some patients could be managed in mini-MDTM/interdisciplinary discussions	◯	◯	◯	◯	◯	◯	◯	◯

15. When your team can not reach a treatment recommendation, which are the most common barriers? (Choose maximum **3** alternatives)

- ☐ Insufficient pathology
- ☐ Insufficient radiology
- ☐ Need for supplementary investigations
- ☐ Insufficient information on comorbidity
- ☐ Insufficient information on patient preferences
- ☐ Patient has other preferences
- ☐ No professional present has seen the patient
- ☐ Absence of key professionals
- ☐ Disagreement
- ☐ Complex cases
- ☐ Insufficient teamwork
- ☐ Insufficient leadership
- ☐ Insufficient preparations
- ☐ Lack of time
- ☐ Interruption or distraction
- ☐ Don’t know
